# Exploring the Effects of Nano-CaCO_3_ on the Core–Shell Structure and Properties of HDPE/POE/Nano-CaCO_3_ Ternary Nanocomposites

**DOI:** 10.3390/polym16081146

**Published:** 2024-04-19

**Authors:** Wei Liu, Lumin Wang, Xun Zhang, Hongliang Huang, Yongli Liu, Minghua Min

**Affiliations:** 1 Key Laboratory of Oceanic and Polar Fisheries, Ministry of Agriculture and Rural Affairs, East China Sea Fisheries Research Institute, Chinese Academy of Fishery Sciences, Shanghai 200090, China; 18817771740@163.com (W.L.); wanglm@ecsf.ac.cn (L.W.); zhangxun007@hotmail.com (X.Z.); ecshhl@163.com (H.H.); 1981-lyl@163.com (Y.L.); 2 Qingdao Marine Science and Technology Center, Qingdao 266237, China

**Keywords:** core–shell structure, HDPE, POE, nano-CaCO_3_, ternary nanocomposites, mechanical properties

## Abstract

To address the dilemma of the stiffness and toughness properties of high-density polyethylene (HDPE) composites, titanate coupling agent-treated CaCO_3_ nanoparticles (nano-CaCO_3_) and ethylene–octene copolymer (POE) were utilized to blend with HDPE to prepare ternary nanocomposites via a two-sequence-step process. Meanwhile, a one-step process was also studied as a control. The obtained ternary nanocomposites were characterized by scanning electron microscopy (SEM), Advanced Rheometrics Expansion System (ARES), Dynamic Mechanical Analysis (DMA), wide-angle X-ray diffraction analysis (WXRD), and mechanical test. The SEM results showed one or two CaCO_3_ nanoparticles were well-encapsulated by POE and were uniformly dispersed into the HDPE matrix to form a core–shell structure of 100–200 nm in size by the two-step process, while CaCO_3_ nanoparticles were aggregated in the HDPE matrix by the one-step method. The result of the XRD showed that the nano-CaCO_3_ particle played a role in promoting crystallization in HDPE nanocomposites. Mechanical tests showed that the synergistic effect of both the POE elastomer and CaCO_3_ nanoparticles should account for the balanced performance of the ternary composites. In comparison with neat HDPE, the notched impact toughness of the ternary nanocomposites of HDPE/POE/nano-CaCO_3_ was significantly increased. In addition, the core–shell structure absorbed the fracture impact energy and prevent further propagation of micro-cracks, thus obtaining a higher notched Izod impact strength.

## 1. Introduction

Polyethylene is the most representative general-purpose polymer among the polyolefin-based polymers. It exhibits properties such as crystallinity and a decreased crystal size depending on the number and length of branches connected to the main chain, and variations can be divided into high-density polyethylene (HDPE) and low-density polyethylene (LDPE). Among them, HDPE has a wide range of industrial applications such as automobiles, electronic appliances, vessels, and tubes [1,2,3,4]. However, HDPE has problems with poorly toughness, strength, and environmental stress cracking resistance [5]. To overcome these disadvantages, polymer blends are prepared by blending and filling to improve the mechanical properties of HDPE. In addition, the incorporation of a rubber filler [6,7], such as the commercial elastomer POE, into HDPE was identified to be an effective method to enhance the toughness of HDPE due to the good biocompatibility between the POE and HDPE. Guimaraes [8] reported that HDPE and POE could be blended together to form composites, and the tensile strength and elongation at break of the composites were clearly improved, which contributes to the good interfacial adhesion between the rubber filler and matrix.

The outstanding properties of inorganic nanoparticles, especially commercial and cheap nanoparticles, are attracting increasing research and market interests, which play important roles in modifying the desirable properties of polymers to form novel functional hybrid materials [9,10]. Ali [11] investigated and determined enhancement of HDPE by choosing nano-calcium carbonate as a typical commercial inorganic nanoparticle to toughen HDPE, but precracks were observed and resulted in a decrease in the fracture toughness of the composites, which probably formed agglomerates of inorganic nanoparticles in the HDPE matrix. With the development of nanotechnology and the hybrid method, the agglomerate problem has been effectively addressed by surface functionalization or modification by functional molecules or polymers; since then, functional inorganic nanoparticles and elastomers have been combined to form ternary polymer blends to toughen HDPE [12,13,14,15,16]. The simple blending process was exploited to fabricate ternary polymer blends with a core–shell morphology by the melt blending method to solve the compatibility problem of inorganic nanoparticles with HDPE and the low stiffness problem of elastomers [17,18]. Additionally, the inorganic–elastomer core–shell structure in ternary blends prepared by melt blending, which combine the properties of inorganic and elastomer building components within a single material, have attracted more and more attention from scientists due to the combination of the robust inorganic blocks and flexible elastomer blocks, which have various functions [19,20,21,22,23,24]. It was revealed that the core–shell particles, which were uniformly dispersed in the matrix, had higher toughening efficiency than common rubber particles. Up to now, the majority of works have focused on how to predict and control the morphologies of the resulting composites, while the properties of the ternary composites are determined not only by the composition and characteristics of the components but also by the phase morphology [17].

In this work, commercial and cheap nano-CaCO_3_ particles and elastomer POE were blended with HDPE to obtain the core–shell morphology HDPE/POE/nano-CaCO_3_ ternary nanocomposites by the two-step melt process. The phase morphology of the ternary composites was predicted by thermodynamic consideration and kinetic factors. The SEM results were compared with the thermodynamic prediction. In addition, the microstructure, dynamic mechanical thermal properties, crystallization properties, and mechanical properties of the ternary nanocomposites were systematically investigated. 

## 2. Materials and Methods

### 2.1. Materials

The HDPE, with a trade mark of 5000S (Yangzi Petrochemical Co., Nanjing, China), a melt flow index of 0.9 g/10 min (190 °C, 2.16 kg), and average molecular weight of 5.28 × 10^5^ g/mol, was supplied in pellet form. POE, the polyolefin elastomer used in this work, was an ethylene-octene copolymer (Engage 8200, Du Pont-Dow Chemical, New York, NY, USA) consisting of 24 wt% octene with a melt flow rate (MFR) of 5 g/10 min (190 °C, 2.16 kg). Nano-sized calcium carbonate (nano-CaCO_3_; average diameter of 60 nm) surface coated with a coupling agent of organic titanate was produced by Guangdong Enping Chemical and Engineering Co., Jiangmen, China.

### 2.2. Specimen Preparation

Two processing methods were applied in the work to prepare the HDPE/POE/nano-CaCO_3_ ternary composites. For the one-step process, the POE and nano-CaCO_3_ particles were directly blended with HDPE at 240 °C and 60 rpm for 8 min in a Haake Rheocorder (Haake PolyDrive, Thermo Scientific, Karlsruhe, Germany). In the two-step process, POE and nano-CaCO_3_ were first mixed at a temperature of 200 °C for 8 min in a two-roll mixer, and then the master batch was blended with the pure HDPE at a temperature of 240 °C for 8 min in the Haake Rheocorder. In addition, the HDPE/POE and HDPE/nano-CaCO_3_ binary blends were also melt blended in the Haake Rheocorder at a temperature of 240 °C. Eventually, the composites were broken and injected into standard bars for mechanical tests with an injection molding machine (SZ-5-C, Shanghai Dehong Machine Co., Ltd., Shanghai, China) at 240 °C. Before melt mixing, all the materials were completely dried in a vacuum oven at 60 °C for 12 h. All composites’ weight ratios for this study are listed in Table 1.

### 2.3. Characterizations

The phase structures of the HDPE nanocomposites were observed by SEM (Quanta450, FEI, Hillsboro, OR, USA). The specimens were obtained by cryogenic fracture. To improve the contrast between the HDPE matrix and elastomer phases, the fractured surfaces were etched by heptane at 50 °C for 5–10 min in advance.

Contact angles were measured in a sessile drop mold with a DSA100 (KRUSS, Hamburg, German). HDPE and POE samples were compression molded between clean silicon wafers at 240 °C for 3 min and then cooled to 25 °C under pressure for 1 min. The nano-CaCO_3_ powders were compression molded with a special mold at 25 °C under a certain pressure. The contact angles were measured using two different liquids (water, diiodomethane), and the mean values of five replicates were recorded.

The rheological testing was carried out using the Advanced Rheometrics Expansion System (ARES TA Instrument, New Castle, DE, USA) instrument. All samples had parallel plates of 25 mm diameter and about 1 mm thickness at 240 °C under a nitrogen environment. The rheological measurements were performed in a frequency range of 102 to 10-1 rad/s.

Dynamic Mechanical Analytical (NETZSCH 242C, Selb, Germany) measurements were performed on the samples of 40 × 12 × 3.2 mm^3^ in size. The temperature ranged from −170 °C to 120 °C at a constant heating rate of 3 °C/min and a frequency of 1 Hz.

The crystal structures of all aggregates were characterized by WAXD (Rigaku D/Max-2550 PC, Tokyo, Japan) using Cu Kα radiation in the 2θ° range of 0–60°. The accelerating voltage was 40 kV, and the tube current was 150 mA. All measurements were performed at room temperature.

The tensile tests were performed in a universal testing machine (Zwick Z100, Ulm, Germany) according to ASTM D-638 (the dog-bone-shaped specimens were 3.2 mm wide and 1.65 mm thick. Five composite specimens were analyzed using a cross-head speed of 10 mm·min^−1^; the test was performed at 25 ± 3 °C.

Impact tests were obtained in accordance with ASTM D256 standards at room temperature in an impact tester (PTM7151-C, Socmc Company, Shanghai, China). The notches (depth of 2.54 mm and radius of 0.25 mm) were machined after injection molding. For each test, five specimens were measured, and the average value was taken.

## 3. Results and Discussion

### 3.1. Prediction of the Phase Morphology

The phase morphology has an important effect on the performance of the polymer blends, and the method to thermodynamically predict the phase morphology of the immiscible ternary systems is the wetting coefficient parameter, which employs interfacial tensions between the components to assess the final thermodynamically preferable morphology. The contact angles of the different materials with water and diiodomethane and the surface tension, dispersion, and polar components of the materials were calculated by Equations (1) and (2) [25] and are listed in Table 2.

(1) $$(1+{cos\theta }_{H_{2}O})\gamma_{H_{2}O}=4\left(\frac{\gamma_{H_{2}O}^{d}\gamma^{d}}{\gamma_{H_{2}O}^{d}+\gamma^{d}}+\frac{\gamma_{H_{2}O}^{p}\gamma^{p}}{\gamma_{H_{2}O}^{p}+\gamma^{p}}\right)$$

(2) $$\left(1+{cos\theta }_{{CH}_{2}I_{2}}\right)\gamma_{{CH}_{2}I_{2}}=4\left(\frac{\gamma_{{CH}_{2}I_{2}}^{d}\gamma^{d}}{\gamma_{{CH}_{2}I_{2}}^{d}+\gamma^{d}}+\frac{\gamma_{{CH}_{2}I_{2}}^{p}\gamma^{p}}{\gamma_{{CH}_{2}I_{2}}^{p}+\gamma^{p}}\right)$$

where $\gamma =\gamma^{d}+\gamma^{p}$, $\gamma_{H_{2}O}=\gamma_{H_{2}O}^{d}+\gamma_{H_{2}O}^{p}$, $\gamma_{{CH}_{2}I_{2}}=\gamma_{{CH}_{2}I_{2}}^{d}+\gamma_{{CH}_{2}I_{2}}^{p}$, γ is the surface tension, d is the dispersion component, p is the polar component, and $\theta_{H_{2}O}$ and $\theta_{{CH}_{2}I_{2}}$ are the contact angles of the polymer with water and diiodomethane, respectively. The interfacial tension of each pair of contacting components can be calculated from surface tension data using the geometric mean equation by Wu [25], which is given as follows:

(3) $$\gamma_{AB}=\gamma_{A}+\gamma_{B}-4\left(\frac{\gamma_{A}^{d}\gamma_{B}^{d}}{\gamma_{A}^{d}+\gamma_{B}^{d}}+\frac{\gamma_{A}^{p}\gamma_{B}^{p}}{\gamma_{A}^{p}+\gamma_{B}^{p}}\right)$$

where $\gamma_{AB}$ is the interfacial tension and $\gamma_{A}$ and $\gamma_{B}$ are the surface tensions of the two contacting components in the blends. Moreover, the wetting coefficient ($\omega_{\alpha }$) can be used to predict the dispersion state in the ternary blends, and it can be calculated from the interfacial tensions in the blends using Young’s equation, as follows:

(4) $$\omega_{\alpha }=\frac{\gamma_{CaCO_{3}-POE}-\gamma_{CaCO_{3}-HDPE}}{\gamma_{HDPE/POE}}$$

where $\omega_{\alpha }$ is the wetting coefficient and $\gamma_{CaCO_{3}-POE}$ and $\gamma_{CaCO_{3}-HDPE}$ are the interfacial tension of CaCO_3_ with POE and HDPE, respectively. 

In general, when $\omega_{\alpha }$ > 1, the nano-CaCO_3_ particles tend to distribute in the HDPE matrix. When −1 < $\omega_{\alpha }$ < 1, the nano-CaCO_3_ particles tend to localize at the HDPE/POE interface, and if $\omega_{\alpha }$ < −1, the nano-CaCO_3_ particles tend to distribute within the POE phase. The calculated interfacial tensions and $\omega_{\alpha }$ are shown in the Table 3. It was found that the final phase structure of the HDPE/POE/nano-CaCO_3_ ternary nanocomposites was predicted to be the core–shell phase morphology in which the nano-CaCO_3_ particles were encapsulated in the dispersed POE phase [26,27,28].

### 3.2. Effect of the Processing Method of the HDPE Nanocomposite Phase Structure

**Figure 1:**
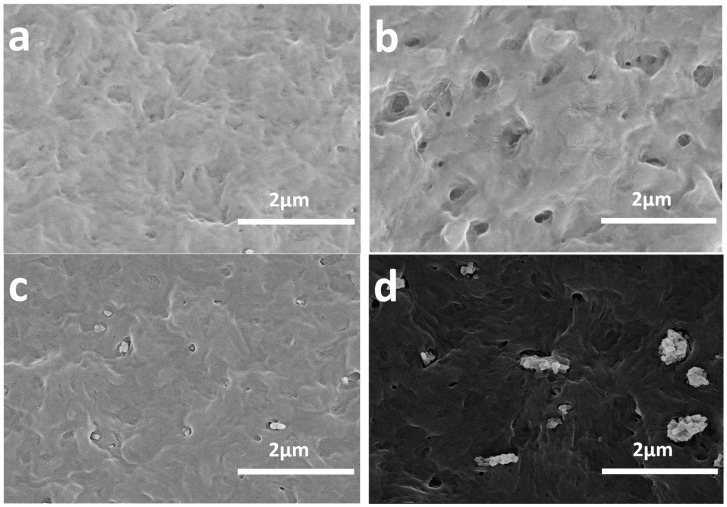
SEM micrographs of cryogenic fractured surface of (**a**) H, (**b**) HP, (**c**) HC5P, and (**d**) HC5P-a.

Ternary composites with a selectively located filler showed poor mechanical properties and low ductility for the weak interface between the two phases in a previous report [29]. To explore the intrinsic enhancing mechanism, SEM images of HDPE composites with different POE and nano-CaCO_3_ contents were investigated. Figure 1 shows the SEM images of the HDPE nanocomposites. All samples were etched with heptane for 8 min at 50 °C to selectively dissolve the POE phase. Thus, the dark holes represented the POE phase in the composites. For the pure HDPE, there were no dark holes, as shown in Figure 1a. However, for the HDPE/POE composite, as shown in Figure 1b, the two-phase morphology was clearly visible in HDPE/POE blend. The dark holes represent POE particles, which were dissolved out by heptane etching. With respect to their sizes, these separated phases were about 200 nm, suggesting good compatibility between HDPE and POE. In Figure 1c, it is clear that the core-shell structures’ domain sizes were quite small (100–200 nm) and had one or two nano-CaCO_3_ particles (60–120 nm) in each hole. This shows that the typical core–shell structure well dispersed in HDPE, which was consistent with the thermodynamic prediction results.

However, in Figure 1d, the composites produced by the one-step process showed that both POE and the majority of the nano-CaCO_3_ particles were separately and directly dispersed in the HDPE matrix. Moreover, the agglomeration of CaCO_3_ nanoparticles was more severe, and only a small amount of core-shell structure was produced, which was different from the result of the thermodynamic prediction. The phenomena might be ascribed to the kinetic factors, such as the blending sequence, diffusion process, and blending time, playing determinative roles in the formation of the composites phase structure in the one-step method. Moreover, not only thermodynamic factors but also kinetic ones should be considered in the morphological evolution of the HDPE/POE/nano-CaCO_3_ system [18,22].

### 3.3. Effect of the Content of the Nano-CaCO_3_ of the Nanocomposite Phase Structure

**Figure 2:**
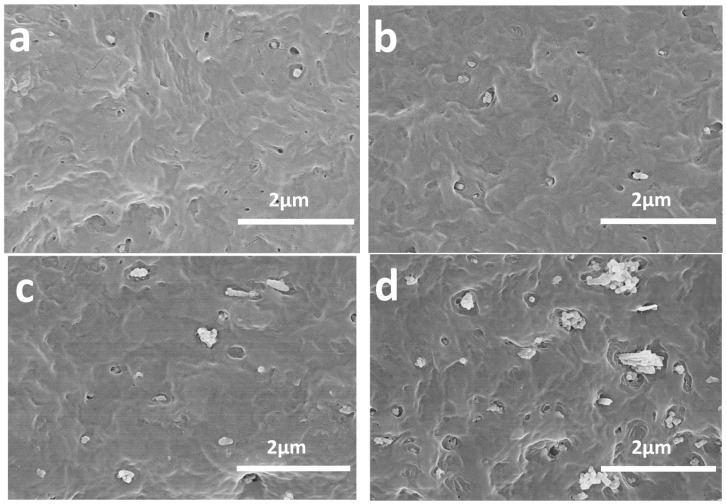
SEM micrographs of cryogenic fractured surface of (**a**) HC1P, (**b**) HC5P, (**c**) HC10P, and (**d**) HC15P.

Figure 2 shows the typical SEM micrographs of the nanocomposites prepared by the two-step process with different nano-CaCO_3_ contents. When POE was mixed with nano-CaCO_3_ in advanced, an encapsulation structure of POE surrounding the nano-CaCO_3_ particles was achieved. The voids around the filler particles unambiguously indicated that the elastomer was located at that place. It is interesting to note that the holes sizes were 100–200 nm, implying that only one to two nano-CaCO_3_ nanoparticles (60–120 nm) were included in the POE phases (Figure 2a). If a longer etching time was applied, the nano-CaCO_3_ particles had to be completely removed together with POE, leaving empty holes in the matrix. With a rise in the content of nano-CaCO_3_ particles, isolated nano-CaCO_3_ particles appeared in the HDPE matrix (Figure 2b,c), but POE-encapsulated nano-CaCO_3_ still represented the typical phase structure. However, when the content of the nano-CaCO_3_ increased further (Figure 2d), a large amount of nano-CaCO_3_ particles agglomerated in the HDPE matrix, which would have a negative effect on the mechanical properties of the nanocomposites. It was observed that the optimum content of HDPE/POE/nano-CaCO_3_ ternary nanocomposites was HC5P.

**Figure 3:**
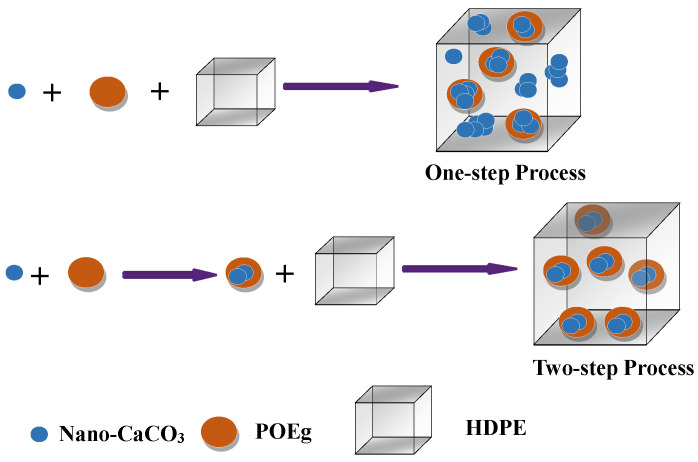
Possible schematic diagram for the formation of the composite phase structure.

Therefore, a possible schematic diagram for the formation of the composite phase structure is shown in Figure 3. It should be noted that both the thermodynamic and kinetic factors should be taken into account. In addition, the thermodynamic factors usually include interfacial tensions between the components, and the kinetic factors are the blending sequence, diffusion process, blending time and so on. It is evident that mixing POE and nano-CaCO_3_ in advanced could produce a unique core–shell structure of POE surrounding the nano-CaCO_3_ particles. Moreover, this distinctive phase structure would directly affect the properties, especially mechanical properties, such as the toughness and strength of the nanocomposites.

### 3.4. Melt Rheological Behavior

**Figure 4:**
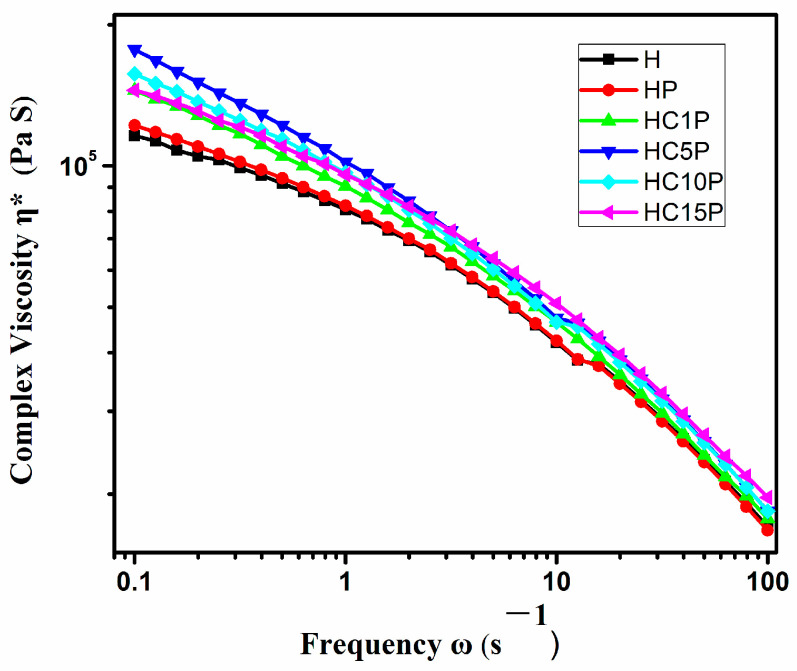
Plots of complex viscosity (η*) with frequency (ω) at 180 °C of the HDPE, HDPE/POE blend, and HDPE/POE/nano-CaCO_3_ composites.

The different domain size of the core-shell structure in the nano-composites by the addition of the nano-CaCO_3_ or POE might be attributed to the viscosity of the composites. Figure 4 shows that HDPE nanocomposites with different amounts of nano-CaCO_3_ presented similar flow behavior in the whole frequency range analyzed. Obviously, the effect of the nano-CaCO_3_ particle amount on the complex viscosity was quite prominent at a low frequency and decreased with increasing frequency because of the shear thinning function [30]. However, the decrease in the core-shell structures’ domain size (D) dispersed in the HDPE nanocomposites might be due to the increased viscosity of the matrix phase (HDPE), as the result was consistent with scanning electron microscopy. The melt viscosity of any system depends on the polymer chain and is affected by even a slight change in the phase morphology of the nanocomposites [31].

### 3.5. Dynamic Mechanical Properties of Composites

**Figure 5:**
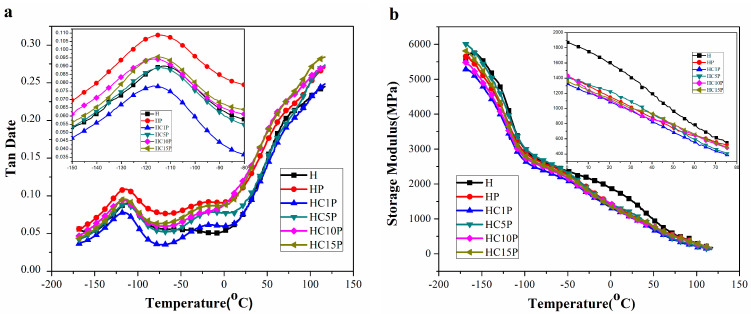
Tanδ and storage modulus of the HDPE, HDPE/POE blend, and HDPE/POE/nano-CaCO_3_ composites (the inset were the locally enlarged image of a certain temperature range).

DMA was used to provide information on the mechanical behavior, molecular relaxations, and interactions taking place in the produced materials as the temperature varied [32]. To study the thermo-mechanical properties of HDPE nanocomposites, the variation of loss factor (tan δ) and storage modulus (E’) with temperature are shown in Figure 5. The damping property of the material provided a balance between the elastic phase and viscous phase in the polymeric structure. The peak of tan δ in Figure 5a indicated the glass-transition temperature (Tg) of the blends. The tan δ peak of HDPE composites occurred around a temperature of −115 °C, which is associated with the glass-transition temperature of HDPE. The peak of tan δ around a temperature of −25 °C is the glass-transition temperature of POE.

The storage modulus (E’) is closely related to the load-bearing capacity of a material [33]. As shown in Figure 5b, as the amount of nano-CaCO_3_ increased, the storage modulus of the HDPE nanocomposites decreased gradually, especially in the range of −50 to 50 °C. It should be noted that the storage modulus of HDPE nano-composites was lower compared to that of pure HDPE. However, the storage modulus of HDPE with different contents of POE/nano-CaCO_3_ composites decreased remarkably, probably due to the increase in the stiffness of the polymer matrix with the reinforcing effect of the shell-core structure, which allowed for a greater degree of stress transfer at the interface [34]. In addition, the storage modulus in the solid state was related to the mechanical and impact strengths of the composites [35].

### 3.6. Wide-Angle X-ray Diffraction

**Figure 6:**
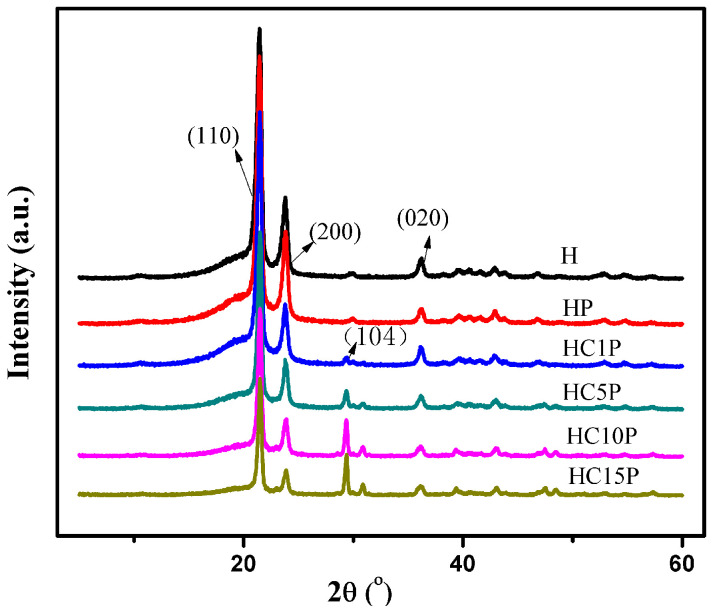
Wide X-ray diffraction patterns of the HDPE, HDPE/POE blend, and HDPE/POE/nano-CaCO_3_ composites.

Wide X-ray diffraction was widely used to study the structure, orientation, crystallinity, and size of the crystallization. The crystal structures of polyethylene were found to be orthorhombic, monoclinic, and hexagonal, depending on the processing conditions reported [23]. X-ray diffraction patterns for HDPE with different nano-CaCO_3_ contents are shown in Figure 6. Three visible crystal diffraction peaks can be seen at around 2θ = 21.6°, 23.8°, and 36.2° in both HDPE and the HDPE composites, which corresponds to the crystal plane (110, 200, and 020) of HDPE [36,37]. Another crystal diffraction peak position was around 2θ = 29.4° in the composites, which corresponds to the crystal plane (104) of calcite [38]. Obviously, the XRD profile of the composites was almost similar to that of HDPE, and the peak position did not change at all. However, the intensity and width of each peak was different, suggesting that there was a change in crystallinity and crystal size in the samples.

According to this, Scherer [39] derived the relationship of the crystal size and the width of the diffraction line:

$$L_{\mathrm{hk}l}=\frac{K\lambda }{\beta \cos\theta }$$

where L_hkl_ is the crystal size perpendicular to the (hkl) plane, θ is the angle between the incidence X-ray and the plane perpendicular to the (hkl) plane, λ is the wavelength of the X-ray (0.1542 nm here), β is the width of the diffraction peak at its half height, and K is the crystal shape factor (0.89 here).

In Table 4, it was found that with the increase in the content of nano-CaCO_3_, the crystallinity of the composites increased because the nano-CaCO_3_ particle plays a role in promoting the crystallization of the HDPE composites.

### 3.7. Mechanical Properties

The processing method affected the morphology of the composites, as shown in Figure 1, and the morphology also affected the mechanical properties of the composites [40]. It was noted that the composites prepared by the two-step process exhibited improved strength and toughness in comparison with the pure HDPE, as shown in Table 5. It was found that the strength and toughness of HDPE were higher than that of the ternary composites prepared by the one-step process. However, the strength and toughness of HDPE were lower than that of the ternary composites prepared by the two-step process. These results show that the core–shell structure was a key factor for strengthening and toughening HDPE through synergetic action.

**Figure 7:**
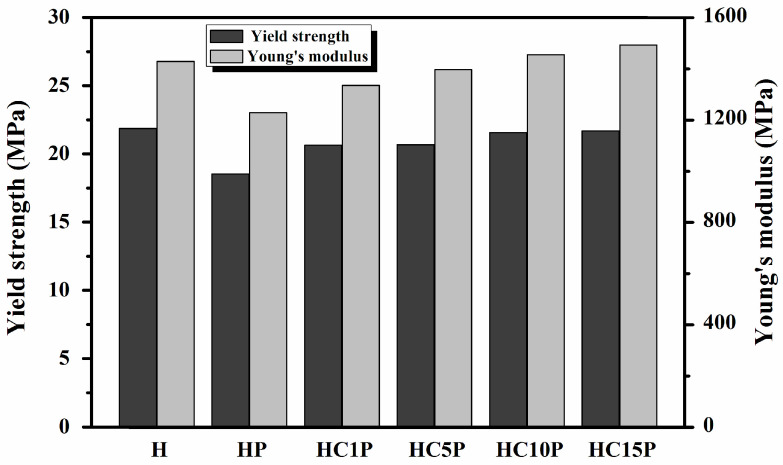
Yield strength and Young’s modulus of the HDPE, HDPE/POE blend, and HDPE/POE/nano-CaCO_3_ composites.

**Figure 8:**
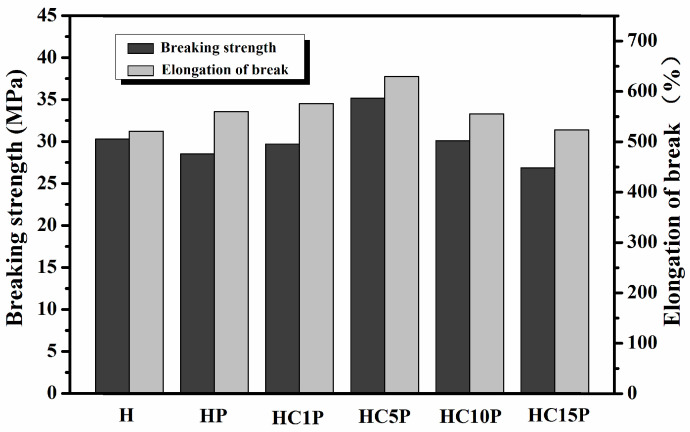
Breaking strength and elongation at break of the HDPE, HDPE/POE blend, and HDPE/POE/nano-CaCO_3_ composites.

Research has shown that the addition of a low-modulus toughening agent improves the impact strength and decreases the tensile and flexural moduli [2]. The tensile properties of ternary composites with different nano-CaCO_3_ contents are shown in Figure 7 and Figure 8. Compared with pure HDPE, both the tensile modulus and tensile strength decreased with the addition of POE. The tensile modulus and tensile strength of the HDPE were 21.87 MPa and 1429.31 MPa, respectively, which were higher than those of the HC1P. However, as the amount of nano-CaCO_3_ increased, the tensile modulus and tensile strength of the HDPE nanocomposites continuously increased, which indicates that nano-CaCO_3_ could improve the stiffness of the composites.

Figure 8 shows the effect of different nano-CaCO_3_ contents on the breaking strength and elongation at break of the HDPE composites. It was found that with the increased amount of nano-CaCO_3_, both the breaking strength and elongation at break first increased and then tended to decrease. When the nano-CaCO_3_ contents were set at a low level (such as HC1P), the breaking strength and elongation at break were 29.70 MPa and 575.75%, respectively. When the nano-CaCO_3_ contents increased, such as in HC5P, the breaking strength and elongation at break reached their maximum, which were 35.17 MPa and 630.08%, respectively. Compared with the pure HDPE, the breaking strength increased by 12.26%, and the elongation at break increased by 20.90%. However, when the nano-CaCO_3_ content was increased further, the breaking strength and elongation at break actually showed a decreasing trend. According to Table 6, as the content of nano-CaCO_3_ in the composites continued to increase, the breaking strength and elongation at break decreased, which could be attributed to the aggregation of the excess nano-CaCO_3_ in the HDPE matrix. Under external forces, the aggregated nano-CaCO_3_ particles were prone to forming cracks in the matrix, thereby reducing the mechanical properties of the composite material, which was consistent with the microstructure of the composites (Figure 3). It was found that the optimum proportion for the mechanical properties of HDPE composites was HC5P.

**Figure 9:**
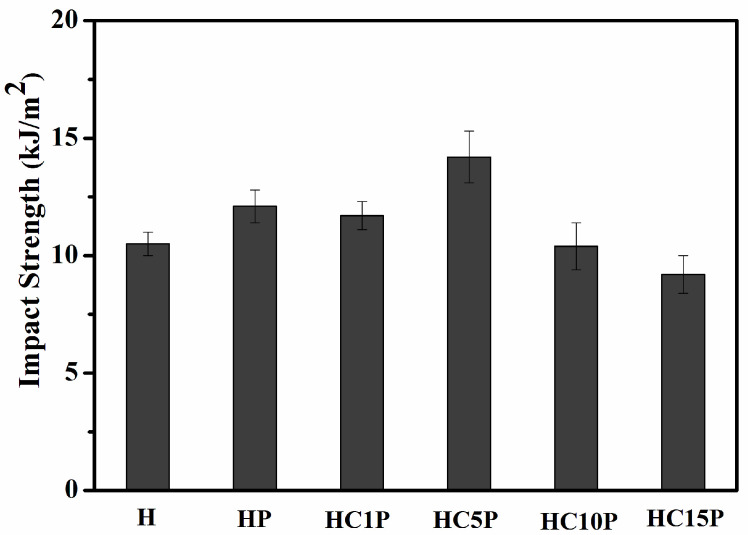
Impact strength of the HDPE, HDPE/POE blend, and HDPE/POE/nano-CaCO_3_ composites.

Generally, polymer blend composites with selectively located fillers showed poor mechanical properties and low ductility because of the weak interface between the two phases. Figure 9 shows the impact strength of the HDPE composites with different nano- CaCO_3_ content. It was found that the addition of POE could increase the toughness of composites. Evidently, the POE-encapsulating CaCO_3_ nanoparticles had the same function as the pure POE in releasing plastic constraints and inducing the shear deformation of the matrix. Compared with pure HDPE, the impact strength of HC5P increased by 35.24%, which was consistent with the results of the elongation at break. From these results, the core–shell structure was a key factor for toughening HDPE through synergetic action, as mentioned above. An elastomer encapsulation with a high affinity for nanoparticle inclusions would favor the absorption of impact energy and prevent the propagation of cracks at the interface [40].

## 4. Conclusions

In this study, elastic (POE) and rigid particles (nano-CaCO_3_) were used as fillers for the preparation of the HDPE composites. According to the SEM and WXRD images, the 100–200 nm core (nano-CaCO_3_) and shell (POE) structure proved to be the key factor, while two-step mixing of the components ensured the POE encapsulated one or two nano-particles. It was found that the core–shell structure played a key role in improving the mechanical properties of HDPE composites, and the HC5P composite had the best comprehensive mechanical properties. The addition of POE and CaCO_3_ nanoparticles into HDPE can bring about significant enhancement of impact toughness without decreasing HDPE’s stiffness and tensile strength.

## Figures and Tables

**Table 1:** Compositions (wt.%) and compounding methods of the blends studied in this work.

Abbreviation	Composition (Weight Ratio)			Processing Method
	HDPE	Nano-CaCO_3_	POE	
H	100	0	0	/
HP	95	0	5	/
HC	95	5	0	/
HC1P	90	1	10	Two-step process
HC5P-a	90	5	10	One-step process
HC5P	90	5	10	Two-step process
HC10P	90	10	10	Two-step process
HC15P	90	15	10	Two-step process

**Table 2:** Contact angle and surface tension of the materials.

Sample	Contact Angle (°)		Surface Tension (mN/m)		
	Water	Diiodomethane	$\mathrm{Dispersion}\;\mathrm{Component}\;(\gamma^{d})$	$\mathrm{Polar}\;\mathrm{Component}\;(\gamma^{p})$	Total (γ)
HDPE	95.7	69.9	19.90	6.70	26.60
POE	106.8	78.4	18.88	2.92	21.80
nano-CaCO_3_	105.2	66.2	26.93	1.32	28.25

**Table 3:** Interfacial tension of each blend and wetting coefficient of the nanocomposites.

Pairs	$\mathrm{Interfacial}\;\mathrm{Tension}\;(\gamma_{AB})$ (mN/m)	$\omega_{\alpha }$	Predictive Morphology
HDPE/POE	1.52	−1.76	Core–shell
HDPE/nano-CaCO_3_	4.69		
POE/nano-CaCO_3_	2.00		

**Table 4:** Crystalline parameters of the HDPE, HDPE/POE blend, and HDPE/POE/nano-CaCO_3_ composites obtained from the XRD curves.

Sample	Crystal Size/nm			Crystallinity (%)
	L(110)	L(200)	L(020)	
H	16.53	14.94	18.54	65.13
HP	17.54	14.34	16.84	60.27
HC1P	20.10	16.77	17.89	60.92
HC5P	21.11	17.09	16.38	69.09
HC5P-a	23.18	16.1	15.79	71.44
HC10P	16.53	14.94	18.54	65.13
HC15P	17.54	14.34	16.84	60.27

**Table 5:** Mechanical properties for the HDPE and HDPE/POE/nano-CaCO_3_ composites with different processes.

Sample	Yield Strength	Tensile Modulus	Breaking Strength	Elongation at Breaking
	(MPa)	(MPa)	(MPa)	(%)
H	21.87 ± 1.12	1429.31 ± 19.27	31.33 ± 1.24	521.17 ± 21.45
HC5P-a	20.42 ± 0.92	1424.56 ± 21.45	28.34 ± 1.42	505.45 ± 19.45
HC5P	22.98 ± 1.22	1435.34 ± 23.05	42.98 ± 0.92	661.76 ± 25.47

**Table 6:** Mechanical properties of the HDPE, HDPE/POE blend, and HDPE/POE/nano-CaCO_3_ composites.

Sample	Yield Strength	Tensile Modulus	Breaking Strength	Elongation at Breaking
	(MPa)	(MPa)	(MPa)	(%)
H	21.87 ± 1.12	1429.31 ± 19.27	31.33 ± 1.24	521.17 ± 21.45
HP	18.53 ± 1.65	1439.25 ± 23.26	28.52 ± 1.50	560.23 ± 22.75
HC1P	20.63 ± 1.24	1406.76 ± 21.57	29.70 ± 1.52	575.75 ± 22.88
HC5P	20.67 ± 1.45	1396.97 ± 20.57	35.17 ± 1.42	630.08 ± 20.74
HC10P	21.56 ± 1.28	1302.45 ± 19.28	30.12 ± 2.75	555.46 ± 24.57
HC15P	21.69 ± 1.54	1269.30 ± 20.84	26.86 ± 1.81	523.92 ± 20.11

## Data Availability

Data are contained within the article.
